# The pattern of mandibular third molar impaction and its relationship with the development of distal caries in adjacent second molars among Emiratis: a retrospective study

**DOI:** 10.1186/s12903-022-02338-4

**Published:** 2022-07-24

**Authors:** Mohammed Amjed Alsaegh, Dana Ayed Abushweme, Khadeija Othman Ahmed, Salhah Othman Ahmed

**Affiliations:** 1 Department of Oral and Craniofacial Health Sciences, College of Dental Medicine, University of Sharjah, P.O. Box: 27272, Sharjah, UAE; 2 Department of Oral and Maxillofacial Surgery, College of Dentistry, Ajman University, Fujairah Campus, Fujairah, UAE; 3 Kalba Specialized Dental Center, Emirates Health Service, MOHAP, Sharjah, UAE

**Keywords:** Impacted teeth, Mandibular third molar, Lower third molar, Wisdom teeth, Classifications of impaction, Impaction, Pattern, Caries

## Abstract

**Objectives:**

The purpose of this study was to investigate the pattern of mandibular third molar (MTM) impaction and associated carious lesions in adjacent mandibular second molars (MSMs) in a sample of Emirati individuals.

**Methods:**

This retrospective study assessed 2000 orthopantomograms of Emirati patients who visited the Specialized Fujairah Dental Center between 2015 and 2020. The depth, ramus relation and angulation of the impacted MTMs were assessed according to the Pell and Gregory classification and Winter’s classification. In addition, carious lesions in adjacent MSMs associated with the evaluated parameters were identified.

**Results:**

A total of 461 (23.05%) of the patients had at least one impacted MTM. The mean age of the study population was 26.24 years. Mesioangular, level B, and class II impactions were the most common, at 47.37% (χ^2^ = 382.134; *p* < 0.001), 45.48% (χ^2^ = 56.889; *p* < 0.001), and 74.05% (χ^2^ = 513.099; *p* < 0.001), respectively. There was a higher percentage of level C impaction among females than among males (χ^2^ = 19.178; *p* < 0.001). A total of 126 impacted teeth (18.36%) had associated carious lesions. These carious lesions were predominantly found in teeth with mesioangular impactions (χ^2^ = 59.430; *p* < 0.001), level A and B impactions (χ^2^ = 23.301; *p* < 0.001), and class II and I impactions (χ^2^ = 17.918; *p* = 0.006).

**Conclusions:**

It is imperative to raise awareness of soft tissue mesioangular-impacted MTMs, as they are the most frequently associated with the development of carious lesions in adjacent MSMs. Approximately one quarter of evaluated Emiratis had at least one impacted MTM, with the most prevalent pattern being class II, level B, and mesioangular impactions. Furthermore, surgical removal is expected to be more challenging for females than for males.

## Introduction

A tooth is considered impacted when its eruption into normal functional occlusion is hindered by other teeth, overlying bone, or soft tissue and it is not fully erupted by its predicted eruption time [1]. Various systemic and local factors contribute to mandibular third molar (MTM) impaction. Genetics, space limitations, physical disruption, inherent defects in the dental lamina, or failure to induce the underlying mesenchyme are some of the factors leading to impaction [2–4]. In a previous meta-analysis of 49 studies involving 83,484 individuals, third molar impaction was found to affect 24.4% of the population. According to this study, the incidence of impacted MTMs was significantly higher than that of impacted upper third molars, and mesioangular impaction was the most prevalent type of impaction, with no differences between the sexes [5]. Due to the high prevalence of impacted MTMs and their associated pathological lesions, the surgical removal of these impacted teeth is one of the most prevalent oral surgeries among young adults.

It is well documented that MTM impactions are associated with many pathologies, including pericoronitis, distal caries, root resorption and distal periodontal pockets of the mandibular second molar (MSM), cysts, and halitosis [6–8]. They can also cause neoplastic changes, orthodontic and prosthetic problems, and temporomandibular joint disorders [9]. Hence, it is recommended to conduct well-defined studies to determine the impaction patterns of MTMs with higher risks of producing pathologies [6]. This will help in deciding whether to remove the impacted lower wisdom tooth as a prophylactic measure.

One of the most common pathological abnormalities associated with impacted MTMs and their adjacent MSMs is caries. Furthermore, the pattern of MTM impaction has an effect on caries development in the MSM [7]. Because of their various spatial positions and relationships with the surrounding anatomical tissues, surgical removal of impacted MTMs is a difficult procedure [4]. There has been evidence that surgical removal of an impacted MTM affects both the pulp sensitivity and the periodontal health of the MSM [10, 11]. Therefore, clinical examination of the MSM is required before and after surgical removal of an impacted MTM [10].

There have been several approaches to assessing MTM impaction. Based on the depth of the impaction relative to the adjacent MSM and the available space between the distal surface of the MSM and the anterior border of the ramus, Pell and Gregory's classification [12] remains widely used in clinical practice. Thus, there are ramus relationships of classes I, II, and III. Furthermore, Pell and Gregory [12] classified the level of impaction based upon the relationship between the occlusal surface of the MTM and the adjacent MSM as A, B, or C. The familiar Winter's classification [13] was based on the angulation of the impacted lower wisdom tooth to the longitudinal axes of the adjacent MSM.

Different ethnic and racial groups may have different patterns of third molar impactions [14]. Accordingly, a variety of studies have been conducted to identify the pattern of impacted MTMs in many countries and different regions within a country. To the best of our knowledge, this is the first study to assess the pattern of MTM impaction among Emiratis. In addition, the associated carious lesions of the impacted MTMs and their adjacent MSMs were investigated. The purpose of these analyses was to support public health services and to determine the potential for prophylactically removing the impacted MTMs.

## Methods

This retrospective study was conducted from February 2020 to February 2021. In this study, 2000 orthopantomograms (OPGs) of Emirati patients who visited Fujairah Specialized Dental Center in the United Arab Emirates (UAE) seeking a variety of dental treatments were reviewed. Individual OPGs acquired between 2015 and 2020 were randomly selected. This study adhered to the principles of the Declaration of Helsinki and was approved by the Research Ethical Committees of Ajman University (Reference No.: D-S-H-19-10-02) as well as the Ministry of Health and Prevention of the UAE (Reference No.: MOHAP/DXB-REC/JFF/No. 14/2020). These two committees waived the need for participant consent for this retrospective study. Out of the 2000 OPGs, 461 showed at least one impacted MTM. Consequently, a total of 686 impacted MTMs were analyzed. In this study, the estimated sample size was calculated using the online Raosoft sample size calculator [15]. Based on a response rate of 50%, a confidence interval of 99%, and a margin of error of 5%, a total of 664 samples were required for infinite population size. Therefore, this study included a convenience sample of 2000 total investigated samples and 686 impaction samples.

Inclusion criteria for patients with impaction included being of Emirati nationality, being older than 18 years, visiting the Specialized Fujairah Dental Center and having an OPG in the system. Meanwhile, patients with impaction were considered when there was at least one impacted MTM with completely formed roots on the panoramic radiograph. The exclusion criteria included insufficient patient records, no Emirati background, poor-quality radiographs, previous orthodontic treatment, age under 18 years or insufficient root formation of the MTM. Exclusions also included missing adjacent MSMs, the presence of any jaw deformity or trauma that could disrupt the alignment of the dentition, and the presence of congenital diseases or facial syndromes, such as cleidocranial dysostosis and Down syndrome.

**Fig. 1:**
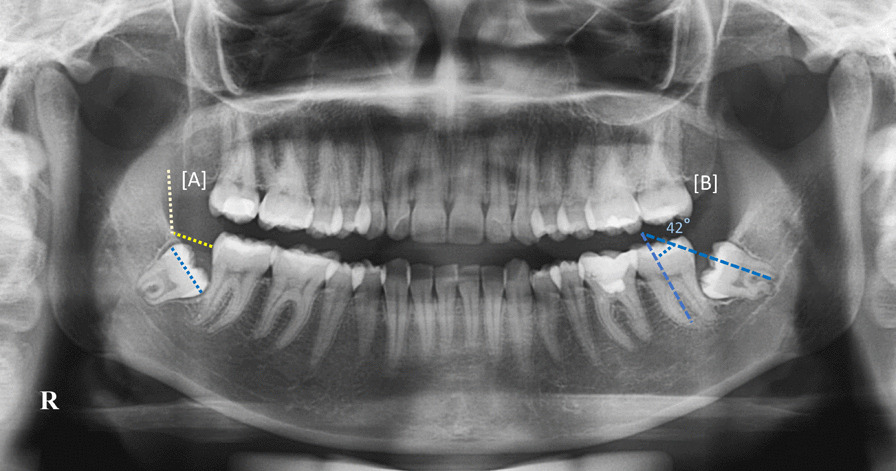
An orthopantomogram (OPG) illustrating the method of measurement used in the study. The impacted lower right third molar is classified as class II and level C according to Pell and Gregory's classification. The long axis of the impacted lower left third molar forms a 42° angle with the long axis of the adjacent second molar. Therefore, it is classified as mesioangular according to Winter’s classification

Digital panoramic radiographs were taken by a MyRay Hyperion X9 (MyRay, Imola-BO, Italy) operated at 5 mA and 76 kV with an exposure time of 9.3 s. Two trained examiners using MyRay software with a magnification of 19% made an independent assessment of the digital OPGs (Fig. 1). Examiners used a protractor provided by the software. Interexaminer error was calculated, and in the case of disagreements between examiners, a final consensus was reached between them.

**Fig. 2:**
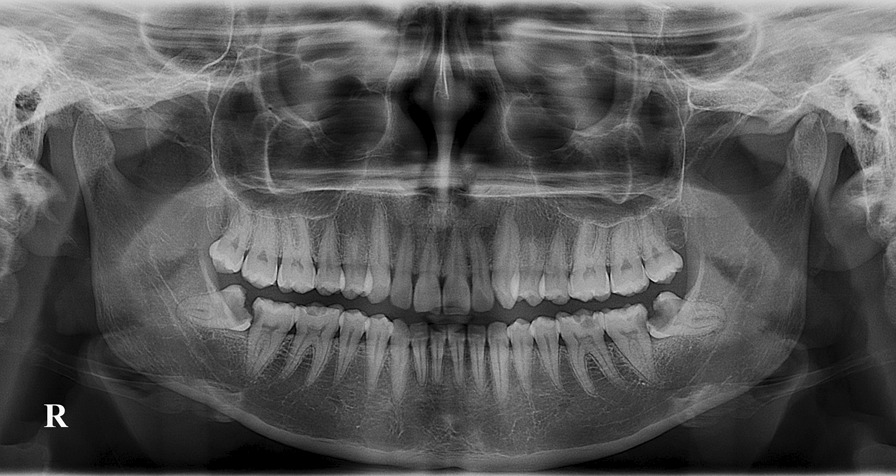
An orthopantomogram (OPG) illustrating the presence of distal caries in both right and left mandibular second molars associated with impacted mandibular third molars

Assessment included the presence and type of impaction as well as associated carious lesions in the MTM and distal caries in the MSM. It was confirmed that the MTM had impaction when it had not fully erupted to the occlusal plane. The relation of the impacted MTM to the ramus was assessed using Pell and Gregory’s classification system [12]. Briefly, class I refers to the condition in which the distance between the distal surface of the MSM and the anterior margin of the mandibular ramus is greater than the anteroposterior dimension of the crown of the MTM. In class II, the distance between the distal surface of the MSM and the anterior margin of the mandibular ramus is smaller than the anteroposterior dimension of the crown of the MTM. Class III is indicated when there is no space between the distal surface of the second molar and the anterior margin of the mandibular ramus. Furthermore, we used the Pell and Gregory classification to determine the level of impaction as follows: level A refers to the occlusal plane of the MTM lying at or above the occlusal plane of the adjacent MSM. The MTM is considered to be level B if its occlusal plane lies somewhere between the occlusal line and the cementoenamel junction (CEJ) of the MSM. Furthermore, level C is described as when the MTM is located below the CEJ of the adjacent MSM. In addition, the angulation of the impacted lower wisdom teeth was determined based on Winter’s classification [13] measured using the method by Quek et al. [16] as follows: When the angle between the long axis of the MTM and long axis of the MSM is between 10° and − 10°, impaction is reported as vertical impaction; if the angle is from 11° to 79°, it is categorized as mesioangular impaction; horizontal impaction and distoangular impaction are defined as angles of 80°–100° and − 11° to − 79°, respectively; and infrequent types of impactions, such as buccolingual and distoinverted, are referred to as “others”. The presence of distal caries in the MSM (Fig. 2) was considered when the OPG showed evidence of distal caries or distal filling with or without related pulpal and periapical disease associated with an adjacent impacted MTM [8].

Statistical Package for the Social Sciences (SPSS) 24.0 software (IBM SPSS, Armonk, NY, USA) was used to analyze the data. The patients’ age, sex, unilateral or bilateral impaction, angulation of impaction, level of impaction, relation of the impaction to the ramus and associated carious lesions are displayed as frequencies and percentages. The chi square test was utilized to detect any possible differences. The results were considered statistically significant if *p* < 0.05.

## Results

A total of 2000 patient records, including OPGs, were reviewed to detect MTM impactions. Among them, 461 (23.05%) patients had at least one impacted MTM, totaling 686 MTM impactions. Females accounted for 433 cases (63.1%), while males accounted for 253 cases (36.9%) (χ^2^ = 47.230, *p* =  < 0.001). The study sample had a mean age of 26.24 years (range = 18–51 years), and 97.8% were younger than 40 years old.

**Fig. 3:**
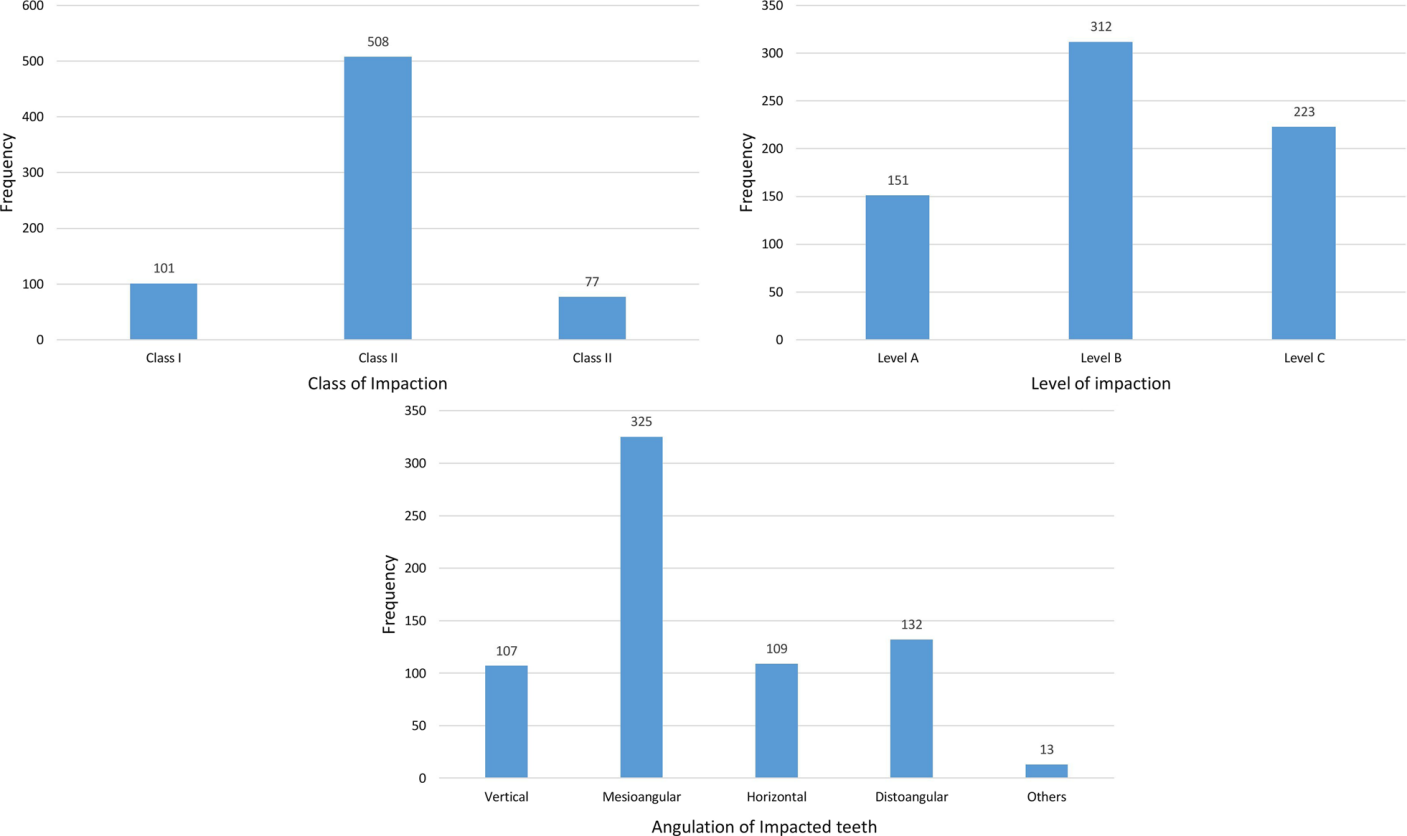
The distribution of impacted lower third molars according to Pell and Gregory and Winter’s classifications

**Fig. 4:**
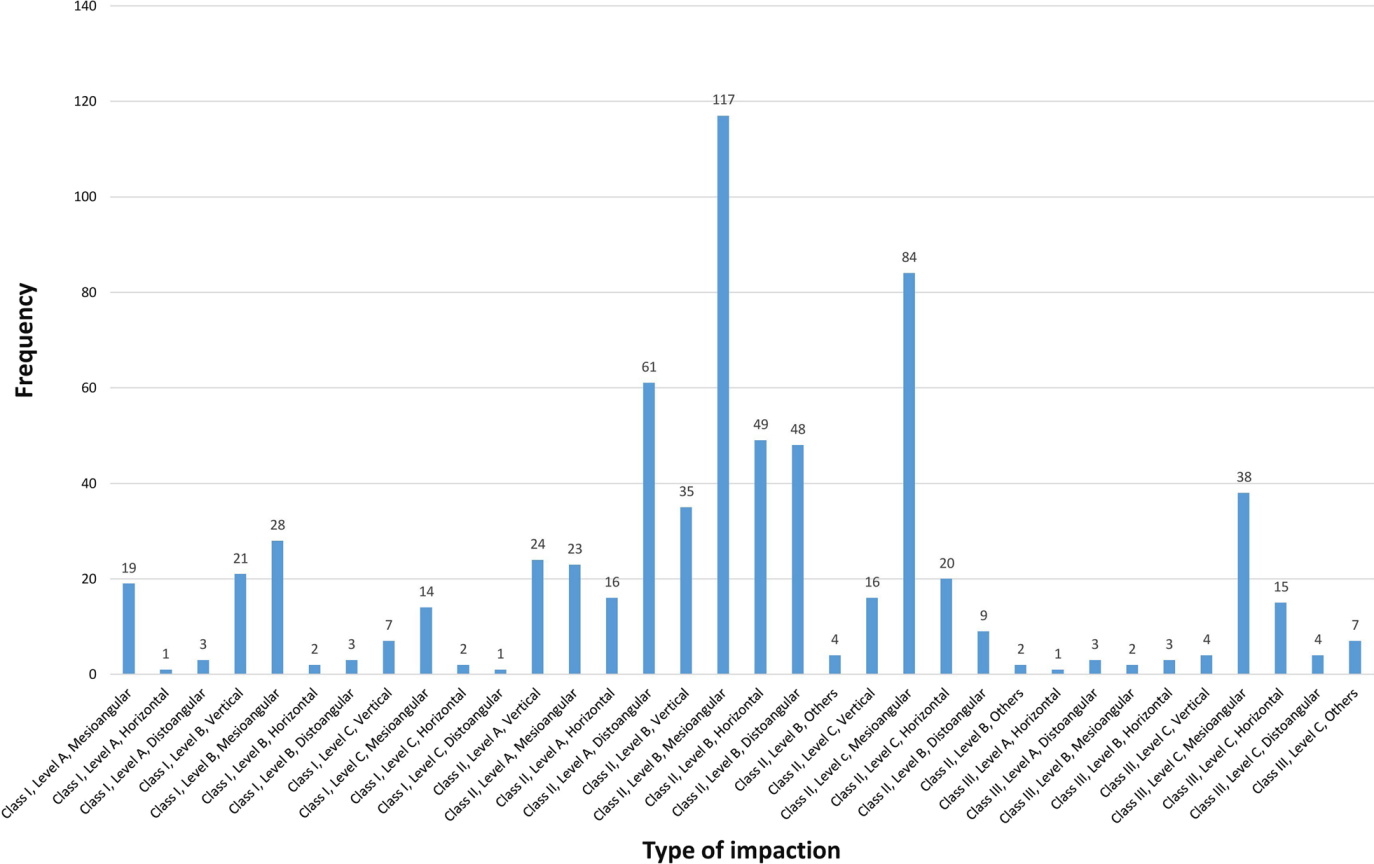
The impaction type of the studied impacted lower third molars according to the Pell and Gregory classification and Winter’s classification

**Fig. 5:**
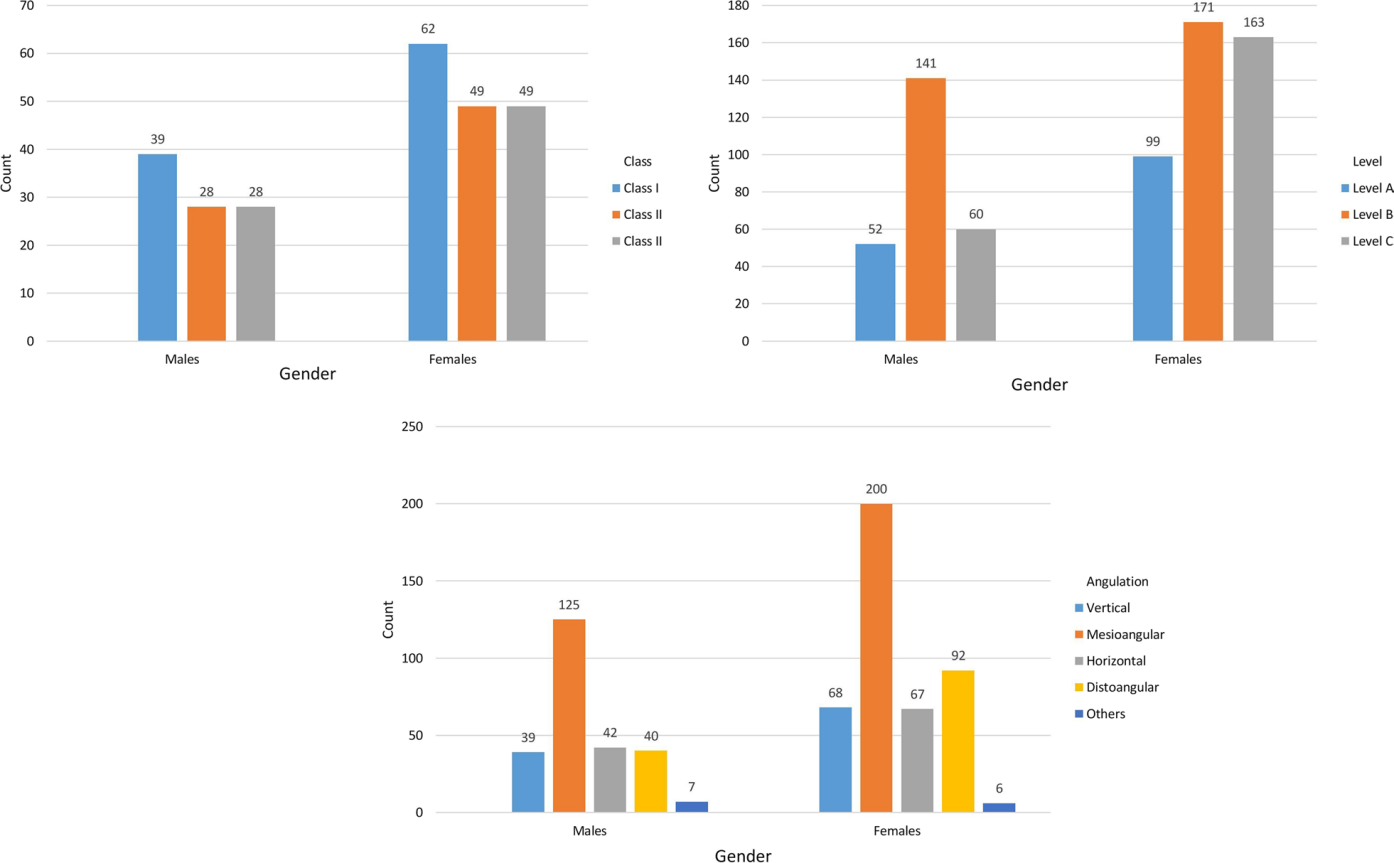
The distribution of impacted lower third molars by sex

There was almost no difference in the impaction side, with 346 and 340 MTM impactions on the left and right sides, respectively. The number of patients with bilateral MTM impactions was 225 (48.80%), whereas the number of patients with unilateral MTM impactions was 236 (51.20%). The most common types of impaction were mesioangular impaction, level B impaction, and class II impaction, at 47.37%, 45.48%, and 74.05%, respectively (Table 1, Figs. 3, 4). Despite a higher percentage of distoangular impaction among females, there was no significant difference in angulation between the two sexes (χ^2^ = 4.546; *p* = 0.337) (Fig. 5). Furthermore, there were no significant differences in the relation of impaction to the ramus between sexes (χ^2^ = 0.155; *p* = 0.926) (Fig. 5). There was, however, a significant difference in the depth of impaction between the two sexes (χ^2^ = 19.178; *p* < 0.001), with females experiencing a higher percentage of level C impaction (Fig. 5).

**Table 1:** The distribution of impacted lower third molars according to the study variables

Variable	Number of impacted tooth (%)	χ^2^	Significance
*Angulation*			
Vertical	107 (15.59%)	382.134	< 0.001
Mesioangular	325 (47.37%)		
Horizontal	109 (15.88%)		
Distoangular	132 (19.24%)		
Others	13 (1.89)		
*Level of impaction*			
A	151 (22.01%)	56.889	< 0.001
B	312 (45.48%)		
C	223 (32.50%)		
*Ramus relationship*			
I	101 (14.72%)	513.099	< 0.001
II	508 (74.05%)		
III	77 (11.22%)		
*Side*			
Right	340 (49.56%)	0.052	0.819
Left	346 (50.43%)

A total of 126 (18.36%) of the impacted MTMs had associated carious lesions (Table 2). Among them, 102 (14.86%) showed distal caries in the adjacent MSM, and 24 (3.49%) showed caries in the impacted MTM. These carious lesions were found mostly in cases of mesioangular impaction (χ^2^ = 59.430; *p* < 0.001), levels A and B impactions (χ^2^ = 23.301; *p* < 0.001), and classes I and II impactions (χ^2^ = 17.918; *p* = 0.006).

**Table 2:** The presence of carious lesions in different lower third molar impactions

Variable	Total	Caries in MSMs	Caries in MTMs	Presence of dental caries in MSMs and MTMs (%)	χ^2^	Significance
*Angulation*						
Vertical	107	5	5	10 (9.34%)	59.430	< 0.001
Mesioangular	325	79	6	85 (26.15%)		
Horizontal	109	12	3	15 (13.76%)		
Distoangular	132	6	10	16 (12.12%)		
Others	13	0	0	0 (0.00%)		
*Level of impaction*						
A	151	23	12	35 (23.17%)	23.301	< 0.001
B	312	54	11	65 (20.83%)		
C	223	25	1	26 (11.65%)		
*Ramus relationship*						
I	101	25	4	29 (28.71%)	17.918	0.006
II	508	73	20	93 (18.30%)		
III	77	4	0	4 (5.19%)		

## Discussion

The surgical removal of impacted teeth is one of the most prevalent oral surgeries among young adults. Indeed, different ethnic and racial groups may have different patterns of third molar impactions [14]. Therefore, studying the pattern of impaction and its related pathological conditions will support public health services and indicate the prophylactic removal of impacted MTMs. To the best of our knowledge, this is the first study in which the pattern of MTM impaction and the resulting carious lesions have been investigated among Emiratis. Studying the pattern of MTM impaction is beneficial for determining the type of anesthesia, duration of the procedure, difficulty of the procedure and possible complications of the surgery [14].

In this report, the prevalence of impacted MTMs was approximately 23%. This prevalence rate draws attention to public health concerns among young adults. A comparable prevalence rate was observed in Saudi Arabia [17]. However, lower [8, 14] and higher [18, 19] prevalence rates have been reported. It is possible that inconsistencies in prevalence may be attributed to the difference in mineralization rates of the MTM, tooth-to-jaw ratios, and physical maturity among the study samples. Furthermore, different sampling methodologies for the same population could affect the results. Consequently, the present study investigated the prevalence of impacted MTMs among Emirati patients attending a governmental dental center in the UAE.

The prevalence of unilateral and bilateral MTM impactions was almost the same in the current study. Comparable results have been observed in a previous study [20]. However, there have been previous reports that showed both higher [14, 16, 18] and lower [8] rates of bilateral impaction.

Level B impaction was the most common level of impaction identified in the present study, a finding that is consistent with several previous studies [2, 17, 18, 20, 21]. However, other previous studies have found a higher level of impaction, where level A was the most common level [3, 4, 6, 14, 22]. The second most common level of MTM impaction in the current study was level C, followed by level A, making the impactions in our sample relatively deeper than those reported in other samples [2–4, 6, 14, 18, 21–23]. Meanwhile, deeper impaction was recorded, with level C being the most prevalent type of impaction in samples from the Malaysian [19] and Turkish [24] populations.

Measuring the angulation of impacted MTMs depends on the angle between the impacted MTM and the adjacent MSM, in which a precise measurement method should be used to obtain the best results. According to the current study, mesioangular impaction is the most common type of impaction. Winter's classification defines mesioangular impaction as when the angle between the impacted MTM and the long axis of the adjacent MSM is between 11 and 79 degrees [13, 16]. During the normal eruption process, the MTM rotates from the horizontal plane to the mesioangular plane and then to the vertical plane. Therefore, mesioangular impaction can be caused by any factor that causes the normal process to fail. It has also been noted that overdevelopment of the mesial root may lead to distoangular impaction, while underdevelopment may result in mesioangular impaction [16].

It is not uncommon to find discrepancies in determining the angle of the impacted MTM in the literature. Some authors employed a visual impression, while others, such as in our study, used a digital protractor that was integrated into the software used. This last method provides more accurate results and allows the methodology of the study to be reproduced. In accordance with our findings, many previous studies have also shown that mesioangular impaction is the most common type of impaction [2–4, 7, 8, 14, 17–21, 23]. On the other hand, some other previous reports demonstrated that the vertical type of MTM impaction is the predominant impaction type [6, 22, 24].

In the current report, the most predominant relationship of the impacted MTM to the ramus was class II, followed by class I and then class III. Several previous studies support this finding [2, 4, 18, 20]. It is possible that discrepancies may occur in all studied parameters, including the angulation, depth, and relative relation to the ramus, due to differences in race and ethnicity of the sample, as well as the use of different sampling methodologies [24]. It is also possible that the different statistical analyses contribute to this controversy.

A higher prevalence of impacted MTMs was observed among females than males in the current study. Previously reported findings are consistent with this finding [4, 17, 21]. This result was attributed to the fact that female bone growth stops earlier than male bone growth. Hence, MTMs erupt where there is possible growth of the mandible in males, but this is not the case in females [1]. In contrast, several previous studies have shown that the prevalence of MTM impaction does not differ by sex [2, 14, 18, 24].

An interesting finding of the current study is that impaction in females is deeper than that in males. Previous studies reported comparable findings [2, 17, 19]. Another important finding of the current study was the higher distoangular impaction rates among females than among males. In two previous studies, higher distoangular impaction rates were also observed among females than among males [17, 19]. Considering the distoangular and deeper impaction in females than in males, it is probable that impacted MTMs in females will be more difficult to remove surgically than those in males.

The incidence of complications related to impacted MTMs appears to be low but is still significant, and the occurrence of these conditions may be influenced by the pattern of impaction [20]. Moreover, some impacted MTMs in relatively older patients show no signs of disease and do not affect adjacent teeth [25]. However, it is still a norm during clinical practice that dentists encounter different types of impacted MTMs with varying pathological conditions within them and in the adjacent MSMs. As these lesions grow slowly, they take years to develop into noticeable symptoms for which the treatment becomes more complex [23]. Among the several associated pathologies, hard tissue disease related to an impacted MTM is primarily related to caries of the adjacent MSM [6].

In the present study, distal caries in MSMs were detected in 14.86% of impactions. Other previous studies have shown comparable percentages of 12.2% [20], 18.75% [6], and 24.63% [23]. Additionally, we found that mesioangular impaction was associated with more distal caries in the adjacent MSM, followed by horizontal impaction. These findings are consistent with previous studies that found equivalent results [6, 7, 26]. Other studies have observed that carious lesions in adjacent MSMs are more associated with mesioangular impaction, followed by distoangular impaction [8, 23]. Additionally, we found that caries were more prevalent in level A and B impactions, as well as in class I and II impactions, than in level C and class III impactions. These findings confirm previous results [7]. Insufficient space for cleaning and improper oral hygiene practices may have contributed to these findings. In addition, this may be due to a poor contact point between the two teeth, which made daily oral hygiene more difficult. A previous study showed that mesioangular and horizontally impacted MTMs grow toward the MSMs, and their cusps make contact with and squeeze the distal surface of the MSM, resulting in distal caries in MSMs [26]. It is obvious that level C implies that the impacted crown is situated below the level of the CEJ of the adjacent MSM, which is normally covered in alveolar bone. As a result, the crown is kept far away from the oral environment and bacterial accumulation. A similar condition could be found in a class III molar relationship, where the crown of the impacted MTM is located in the ramus of the mandible. Considering the relatively high percentage of distal tooth decay in the adjacent MSM, prophylactically removing mesioangularly impacted MTMs may be helpful, especially if they are not completely buried in the bone.

The present study is limited by the fact that it is a cross-sectional study that screened only a limited sample of Emirati patients who visited the Fujairah Specialized Dental Center. Thus, almost all of the patients were suffering from symptoms or pathologies. Additionally, this is a retrospective study, as other pathologically associated lesions, such as pericoronitis and periodontal pockets, can only be identified through clinical examinations. Hence, a community survey with a large sample size or a prospective study will be needed in the future. In addition, a more precise classification based on CBCT examination will be more informative.

## Conclusions

Based on the present study, there is a greater need for awareness and even prophylactic removal of mesioangularly impacted MTMs, particularly if they are not completely buried in the bone. Approximately one quarter of Emiratis have at least one impacted MTM, with the most common pattern being class II, level B, and mesioangular. Furthermore, females are more likely to have impacted MTMs than males, and surgical removal is predicted to be more challenging for females than for males.

## Data Availability

The datasets generated and analyzed during the current study are available in the Mendeley Data Repository, https://doi.org/10.17632/ycz3gwxwv6.1.

## Abbreviations

MTM: Mandibular third molar
MSM: Mandibular second molar
OPG: Orthopantomogram
CEJ: Cementoenamel junction
UAE: United Arab Emirates
